# Multifunctional Phosphorescent Conjugated Polymer Dots for Hypoxia Imaging and Photodynamic Therapy of Cancer Cells

**DOI:** 10.1002/advs.201500155

**Published:** 2015-09-10

**Authors:** Xiaobo Zhou, Hua Liang, Pengfei Jiang, Kenneth Yin Zhang, Shujuan Liu, Tianshe Yang, Qiang Zhao, Lijuan Yang, Wen Lv, Qi Yu, Wei Huang

**Affiliations:** ^1^ Key Laboratory for Organic Electronics and Information Displays and Institute of Advanced Materials (IAM)Jiangsu National Synergetic Innovation Center for Advanced Materials (SICAM)Nanjing University of Posts and Telecommunications (NUPT)Nanjing210023JiangsuP.R. China; ^2^ Key Laboratory of Flexible Electronics (KLOFE) and Institute of Advanced Materials (IAM)Jiangsu National Synergetic Innovation Center for Advanced Materials (SICAM)Nanjing Tech University (NanjingTech)Nanjing211816JiangsuP.R. China

**Keywords:** biosensors, conjugated polymer dots, hypoxia imaging, phosphorescence, photodynamic therapy

## Abstract

Molecular oxygen (O_2_) plays a key role in many physiological processes, and becomes a toxicant to kill cells when excited to ^1^O_2_. Intracellular O_2_ levels, or the degree of hypoxia, are always viewed as an indicator of cancers. Due to the highly efficient cancer therapy ability and low side effect, photodynamic therapy (PDT) becomes one of the most promising treatments for cancers. Herein, an early‐stage diagnosis and therapy system is reported based on the phosphorescent conjugated polymer dots (Pdots) containing Pt(II) porphyrin as an oxygen‐responsive phosphorescent group and ^1^O_2_ photosensitizer. Intracellular hypoxia detection has been investigated. Results show that cells treated with Pdots display longer lifetimes under hypoxic conditions, and time‐resolved luminescence images exhibit a higher signal‐to‐noise ratio after gating off the short‐lived background fluorescence. Quantification of O_2_ is realized by the ratiometric emission intensity of phosphorescence/fluorescence and the lifetime of phosphorescence. Additionally, the PDT efficiency of Pdots is estimated by flow cytometry, MTT cell viability assay, and in situ imaging of PDT induced cell death. Interestingly, Pdots exhibit a high PDT efficiency and would be promising in clinical applications.

## 1 Introduction

Molecular oxygen (O_2_) plays a key role in many vital physiological processes, such as numerous enzymatic transformations, ATP, regulation of ion fluxes, signaling pathway, and oxidative phosphorylation.^1^ Consequently, in the investigation of the presence, viability, metabolic status, and complex physiological activity of living systems, O_2_ could be viewed as a unique informative marker to reflect the condition of physiological processes which consume or release O_2_. The abnormal changes of intracellular O_2_ (icO_2_) levels are usually related to diseases. For example, oxygen deprivation (hypoxia) has been identified as one of the most important features of many diseases, including solid tumors,^2^ cardiovascular diseases,^3^ stroke,^4^ Alzheimer's and Parkinson's diseases.^5^ The center of some solid tumor tissues is absolutely hypoxic (0% O_2_).^6^ Although many efforts have been made for early‐stage diagnosis and treatment of cancers, there are still many difficulties in the medical field.^7^ The icO_2_ level provides an important parameter in the early‐stage diagnosis of cancers.^8^, ^9^, ^10^, ^11^

So far, instrument techniques, including positron emission tomography (PET), functional magnetic resonance imaging (MRI), electron paramagnetic resonance (EPR), and pulse oximetry, have been used for hypoxia imaging in vitro and in vivo.^9^ Optical imaging techniques also play important roles in the icO_2_ detection. Among a variety of emissive dyes available for icO_2_ detection,^10^ phosphorescent transition‐metal complexes (PTMCs)^11^, ^12^ have unique advantages, owing to the efficient reversible quenching of phosphorescence by the triplet ground state of O_2_. In addition, PTMCs exhibit large Stokes shift, high photostability, and long emission lifetime, which are beneficial for biosensing and bioimaging. Furthermore, the signal‐to‐noise ratios in the detection of intracellular O_2_ can be enhanced by using time‐resolved luminescence imaging techniques, which distinguish the long phosphorescence lifetime of PTMCs from the short‐lived background fluorescence interference.^13^

Chemotherapy, radiation, and surgery are commonly used in the clinical application of cancer therapy. However, these clinical therapies often lead to serious damages on healthy tissues. Photodynamic therapy (PDT) technique, in which tumor cells are killed by light‐induced production of ^1^O_2_, receives increasing interest in clinical application for cancer treatment due to its unique advantages,^14^ such as low damage to healthy tissues, efficient therapeutic effect, and controllable and high selectivity for the lesion area. Since PTMCs are excellent photosensitizers for ^1^O_2_ generation,^15^ they are potential candidates for PDT applications.

Over the recent decade, many PTMCs‐based icO_2_ probes and PDT photosensitizers have been reported.^11^, ^12^, ^15^ However, many of the reported probes and photosensitizers are hydrophobic small molecules, which suffer from poor water‐solubility, high cytotoxicity, and low molar absorption coefficient in the visible region. Moreover, most of icO_2_ probes are based on the single‐intensity‐based sensing, which suffers from external influence. The above problems have impeded the biomedical applications of small molecular PTMCs. To solve these problems, hyperbranched conjugated polyelectrolyte containing PTMCs were considered as an excellent platform for simultaneous icO_2_ sensing and PDT due to their advantageous properties, such as good water‐solubility, excellent light‐harvesting and light‐amplifying properties, low toxicity, and their compact 3D topology structure with a large number of functional groups.^16^, ^17^

**Figure 1:**
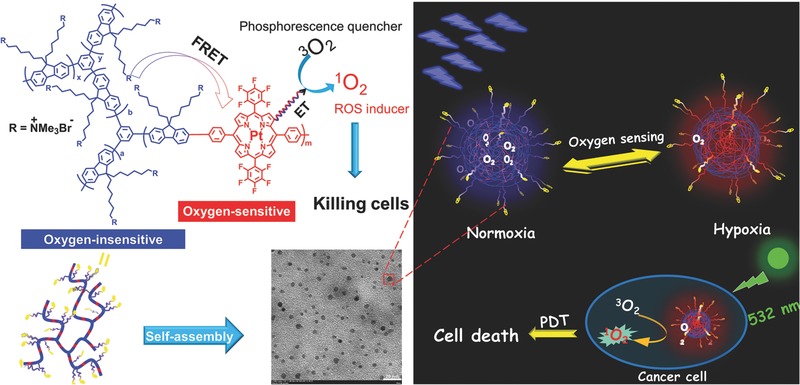
Chemical structures of P2, TEM image (Pdots in PBS solution, scale bar = 20 nm), self‐assembly, O_2_ sensing, and PDT mechanisms of phosphorescent Pdots.

In this study, a kind of water‐soluble and multifunctional phosphorescent polymer dots (Pdots) was designed and synthesized to investigate their icO_2_ detection and PDT for cancer cells. We chose Pt(II) porphyrin as an oxygen‐responsive phosphorescent reagent because of its long luminescence lifetime and high sensitivity toward O_2_, and introduced it into oxygen‐insensitive polyfluorene‐based hyperbranched conjugated polyelectrolyte (**Figure** **1**) to improve the biocompatibility and water‐solubility of Pt(II) porphyrin. Their compact 3D topology structure will benefit to reduce the intermolecular aggregation induced luminescence quenching which exists in linear conjugated polymers.[[qv: 12e‐g]],^16^ Meanwhile, due to the amphiphilic structure, the polyelectrolytes could form ultra‐small polymer dots with a size of ≈10 nm by self‐assembly. Additionally, the ratiometric O_2_ detection could be achieved due to the Förster resonance energy transfer (FRET) from polyfluorene units to Pt(II) porphyrin. Compared with single‐intensity‐based sensing, ratiometric detection, in which the ratio of the intensities at two emission wavelengths is used to reflect the O_2_ levels, shows high resistance against external influence, resulting in more accurate sensing results. Furthermore, the long luminescence lifetime of Pt(II) porphyrin was utilized in photoluminescence (PL) lifetime imaging microscopy (PLIM) and time‐gated luminescence imaging (TGLI). Thus, the interference of short‐lived autofluorescence was eliminated effectively to improve the reliability of imaging. Importantly, as a PDT anticancer reagent for cancer treatment, a highly light‐induced singlet oxygen quantum yield of Pdots in aqueous solutions was determined. The PDT performance of Pdots was investigated by flow cytometry, MTT cell viability assay, and real‐time luminescence analysis of apoptotic cells in situ. Finally, the phosphorescent Pdots–based diagnosis–therapy integrative systems were constructed for cancer treatment. To the best of our knowledge, this is the first time of using phosphorescent Pdots–based system for both icO_2_ detection and PDT of cancer cells.

## 2 Results and Discussion

### 2.1 Design, Synthesis, and Characterization

**Figure 2:**
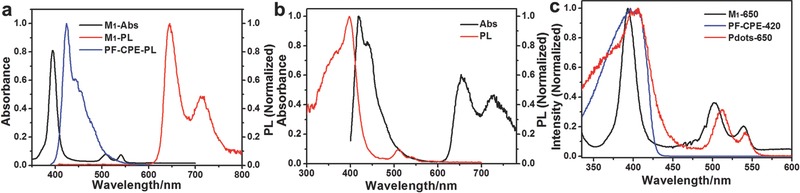
a) Absorption and PL spectra (excited at 375 nm) of 5,15‐bis(pentafluorophenyl)‐10, 20‐bis(4‐bromophenyl) platinum(II) porphyrin (M_1_, 5.6 × 10^–6^ m) in CH_2_Cl_2_ and PL spectrum of pure polyfluorene‐based CPE (14 μg mL^–1^) in PBS solution. b) Absorption and emission spectra of Pdots in aqueous solution (air, *λ*_ex_ = 375 nm, 12 μg mL^–1^). c) Excitation spectra of M_1_ in CH_2_Cl_2_ (M1‐650), Pdots in aqueous at 650 nm (Pdots‐650), and PF‐CPEs in aqueous at 420 nm (PF‐CPE‐420).

Pt(II) porphyrin complexes have been demonstrated to be excellent O_2_ probes and photosensitizers for PDT.^12^ However, their low biocompatibility and poor water‐solubility influence the practical biomedical applications. Herein, we designed and synthesized a polyfluorene‐based hyperbranched conjugated polyelectrolyte containing Pt(II) porphyrin complexes (shown in Scheme S1, Supporting Information), which can be used to ratiometrically and quantitatively detect O_2_ and generate ^1^O_2_ with high efficiency for PDT. The fluorinated phenyl of Pt(II) porphyrin will benefit to improve the solubility and photostability. The characteristic absorption and emission spectra of monomer Pt(II) porphyrin (M_1_) and the emission of pure polyfluorene‐based conjugated polyelectrolyte (PF‐CPE, Figure S1, Supporting Information) were shown in **Figure** **2**a. The strong absorption band at 395 nm was corresponding to the Soret band of Pt(II) porphyrin, and the weak absorption bands at 510 and 540 nm were the Q (1, 0) and Q (0, 0) bands, respectively. Pt(II) porphyrin exhibits a strong emission at 650 nm with a shoulder at 725 nm. Due to the overlap between absorption bands of Pt(II) porphyrin and fluorescence bands of polyfluorene, the FRET process from blue‐emitting and oxygen‐insensitive polyfluorene to red‐emitting and oxygen‐sensitive Pt(II) porphyrin can take place when polyfluorene is excited. Thus, a ratiometric detection of O_2_ can be realized. Moreover, taking advantages of the amplified signal output of conjugated polymer and the FRET process from polyfluorene to Pt(II) porphyrin, sensitive O_2_ sensing could be realized.

The synthetic route of polymer is shown in Scheme S1 (Supporting Information). The polymer precursor was prepared by Suzuki coupling reaction and the target polymer was obtained by the quarternization of the precursor. The polymer was characterized by ^1^H NMR, ^13^C NMR, and GPC. The content of Pt(II) porphyrin complexes in the conjugated polymer was 9.8% (molar ratio) calculated by ^1^H NMR, which was lower than that in the feed ratio probably due to the difference of reaction activity and steric hindrance of Pt(II) porphyrin complexes. The average molecular weight (*M*_w_) of the polymer precursor estimated by GPC was 81 490 g mol^−1^ with a polydispersity index (PDI) of 1.25. Transmission electron microscopy (TEM) was used to study the morphologies of polymer in phosphate buffered saline (PBS) solution (PBS, pH = 7.4, 25 °C). As the TEM image shown in Figure 1, the well‐dispersed Pdots with the size of ≈10 nm were formed through self‐assembly of polymer due to their amphiphilicity and hyperbranched compact structures. The hydrodynamic diameter and average zeta potential of the Pdots were determined by dynamic light scattering (DLS). The mean hydrodynamic diameter of the Pdots is 18 nm and their average zeta potential is 42.2 mV (Figure S2, Supporting Information).

### 2.2 Photophysical Properties

The absorption, excitation, and emission spectra of Pdots, PF‐CPE, and Pt(II) porphyrin monomer have been measured. The effective concentrations of Pt(II) porphyrin in Pdots were calculated based on the ratio of Pt(II) porphyrin, which was characterized by ^1^H NMR. For Pdots, as shown in Figure 2b, the main broad absorption band at 400 nm was attributed to the mixed absorption of polymer backbones (strong π–π* transition) and the Soret bands of Pt(II) porphyrin. The two weak absorption bands at 510 and 540 nm were corresponding to the Q (1, 0) and Q (0, 0) of Pt(II) porphyrin moiety. In addition, the PL spectrum of Pdots has also been investigated (excited at 375 nm). The fluorescence emission peak at 420 nm and a shoulder peak at 440 nm were attributed to the emission of polyfluorene backbone, and the red phosphorescence emission at 650 nm and a shoulder at 725 nm were assigned to the characteristic emission of Pt(II) porphyrin units. Based on the overlap between the emission bands at 420 nm and both of Soret and Q absorption bands of Pdots, the Förster radius is calculated to be 6.6 nm (Supporting Information), indicating that an efficient FRET from polyfluorene to Pt(II) porphyrin could occur. In order to further confirm the FRET process, the excitation spectra of Pt(II) porphyrin, PF‐CPE and Pdots have been investigated, which were shown in Figure 2c and Figure S3 (Supporting Information). The excitation spectral profile (*λ*_em_ = 650 nm) of Pdots was a sum of those of M_1_ and PF‐CPE, indicating the presence of FRET in Pdots. In addition, under different excitation conditions, Pdots always show more efficient FRET than the blend of Pt(II) porphyrin and Pt(II)‐free polymer PF‐CPE according to the absorption and PL spectra shown in Figure S4 (Supporting Information). This will benefit to promote the biocompatibility of Pdots to cells with the reduced dose of lipophilic Pt(II) porphyrin. The luminescence lifetimes of Pdots have also been measured to be 0.74 ns at 420 nm and 11.5 μs at 655 nm.

### 2.3 O_2_ Sensing of Pdots in Aqueous Solution

**Figure 3:**
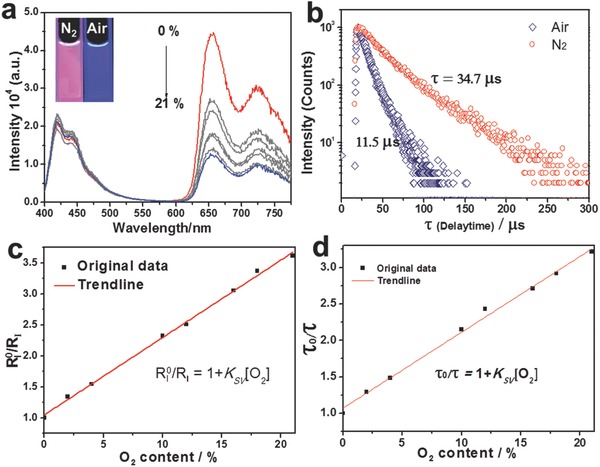
a) Emission spectra of Pdots (12 μg mL^–1^) in the aqueous solution of H_2_O/CH_3_OH = 99/1 under different oxygen contents. *λ*_ex_ = 375 nm. b) Luminescence decays of Pdots at 650 nm in aqueous solution saturated with N_2_, air, and O_2_, respectively. c) Plots of *R*_I_^0^/*R*_I_ as a function of oxygen contents (*R* ^2^ = 0.997). d) Plots of *τ*_0_/*τ* as a function of oxygen contents (*R* ^2^ = 0.995).

Oxygen sensing experiments of Pdots in aqueous solution were carried out. The PL spectra of Pdots under O_2_ contents from 0% to 21% were shown in **Figure** **3**a. The emission bands and lifetimes of polyfluorene backbone at 420 and 440 nm were independent of the change of oxygen contents (Figure S5, Supporting Information). However, the phosphorescence intensity of Pt(II) porphyrin at 650 and 722 nm was very sensitive to O_2_ contents. Under low O_2_ contents, the emission of Pt(II) porphyrin dominates the PL spectrum of Pdots. With the increase of O_2_ content, the emission intensity of Pt(II) porphyrin decreased significantly, and the emission spectrum was dominated by polyfluorene when the contents of O_2_ were up to 21%. Thus, with the blue‐emitting fluorescence from polyfluorene as the reference, Pdots could be an excellent ratiometric probe for sensing O_2_. The large difference between these two emission wavelengths (up to 230 nm) allows accurate measurement of two emission intensities to yield the ratiometric value. Furthermore, the shift of the emission maximum from 420 to 650 nm when the O_2_ contents decreased from 21% to 0% resulted in a clear luminescence color change from blue to red, which could be observed by naked eyes (Figure 3a inset). On the other hand, as shown in Figure 3b, the emission lifetimes at 650 nm were changed from 34.7 to 11.5 μs when the O_2_ contents changed from 0% to 21%. So the O_2_ sensing performance based on emission lifetimes could also be realized. To quantitatively analyze oxygen contents in solutions, the ratios of phosphorescent emission intensity at 650 nm to fluorescence at 420 nm in the absence and presence of oxygen were defined as *R*_I_^0^ = *I*_p_^0^/*I*_f_^0^ and *R*_I_ = *I*_p_/*I*_f_, respectively. The emission lifetimes at 650 nm in the absence and presence of oxygen were defined as *τ*_0_ and *τ*, respectively. The *K*_SV_ value was determined according to the following Stern–Volmer equation

(1) $$\frac{R_{I}^{0}}{R_{I}}=\frac{\tau_{0}}{\tau }=1+K_{\mathrm{SV}}[O_{2}]$$

where [O_2_] was the partial pressure of oxygen. A linear relation between *R*_I_^0^/*R*_I_ and [O_2_], *τ*_0_/*τ* and [O_2_] has been obtained, as shown in Figure 3c. The quenching constant *K*_SV_ was calculated to be 12.5 (1.63 × 10^−2^ mmHg^−1^). In addition, we measured the emission lifetimes at 650 nm with different oxygen contents. The phosphorescence lifetimes also show a linear relation to the oxygen contents (Figure 3d) and give out a *K*_SV_ of 11.4 (1.49 × 10^−2^ mmHg^−1^). This value of *K*_SV_ was in accordance with that determined by luminescent intensity. The good linearity indicated the excellent sensitivity and reliability of Pdots for oxygen quantification.

### 2.4 O_2_ Sensing of Pdots in Solid Film

Next, O_2_ sensing performance of Pdots in solid film was also studied to confirm the reliability of our oxygen measurements. The photoluminescence imaging of Pdots film under different oxygen contents was shown in Figure S6 (Supporting Information). No evident change was observed for the emission intensity from polyfluorene collected at 420–460 nm under different oxygen contents (Figure S6a, Supporting Information (air) and Figure S6b, Supporting Information (2.5% O_2_)). However, the stronger red emission at 630–680 nm (Figure S6c,d, Supporting Information) attributed to Pt(II) porphyrin was observed after reducing oxygen contents. These results indicated that Pdots could be used as an excellent solid‐film oxygen sensor.

Additionally, we demonstrated the oxygen sensing via PLIM and TGLI. From the PLIM images shown in Figure S7a (Supporting Information) (air) and Figure S7b (Supporting Information) (2.5% O_2_), we could see the average emission lifetime of Pdots increased from 877 to 2500 ns when the oxygen contents were reduced from 21% to 2.5%, due to the quenching of phosphorescence of Pt(II) porphyrin by oxygen. To demonstrate the advantage of long phosphorescence lifetime in eliminating short‐lived background interference, we performed time‐gated luminescence imaging (shown in Figure S7c–f, Supporting Information) under air and 2.5% O_2_. A much larger difference in the brightness of the images recorded under air and 2.5% O_2_ was observed when a delay time of 125 ns is exerted (Figure S7e,f, Supporting Information). These results showed a longer lifetime of Pdots after hypoxia. All the above results demonstrated the excellent performance of Pdots as an oxygen sensor in both solution and solid state.

### 2.5 Ratiometric Luminescence Imaging of icO_2_ Levels

**Figure 4:**
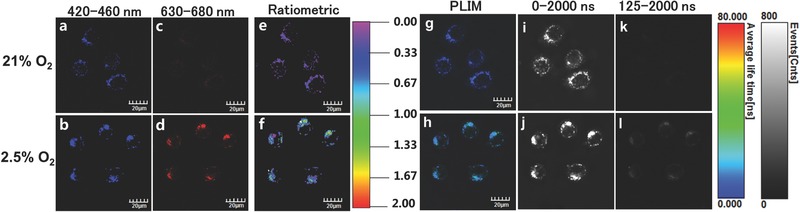
a–f) Photoluminescence images, g,h) photoluminescence lifetime images, and i–l) time‐gated luminescence images with different delay time of HepG2 cells incubated with 7.5 μg mL^–1^ Pdots under different oxygen contents (21% and 2.5% O_2_). Collecting ranges were 420–460 nm for polyfluorene emission and 630–680 nm for Pt(II) porphyrin emission, respectively. Ratio of emission intensity at 630–680 nm to that at 420–460 nm was shown in (e) and (f). *λ*_ex_ = 405 nm.

The performance of Pdots applied for imaging intracellular oxygen levels in HepG2 cells has been investigated. HepG2 cells were cultured under 21% O_2_ atmosphere for 24 h at 37 °C. The Pdots were added to the medium at a concentration of 7.5 μg mL^–1^ and cells were incubated for 2 h under 21% and 2.5% O_2_ contents. We chose 405 nm as the excitation wavelength. From the images shown in **Figure** **4**a–d, we could see that the emission intensity of Pt(II) porphyrin collected at 630–680 nm exhibits an obvious enhancement when the O_2_ content decreases from 21% to 2.5%. In contrast, the emission intensity of polyfluorene at 420–460 nm was almost the same under both 21% and 2.5% O_2_ contents. Furthermore, the real O_2_ levels could be calculated by the ratio of emission intensity at 630–680 nm over that at 420–460 nm. As shown in Figure 4e,f, the value of emission ratio (*I*_red_/*I*_blue_) in living cells changed evidently when cultured at different O_2_ contents, which indicated the excellent ratiometric O_2_ biosensing properties of Pdots nanoprobe.

### 2.6 Time‐Resolved Luminescence Imaging of icO_2_ Levels

Next, we performed the photoluminescence lifetime imaging of HepG2 cells under different oxygen concentrations as shown in Figure 4g (21% O_2_) and Figure 4h (2.5% O_2_). As expected, cells cultured under 2.5% O_2_ displayed a longer lifetime. And the averaged luminescence lifetimes under 21% O_2_ and 2.5% O_2_ were measured to be 25 and 50 ns, respectively. To further utilize the long‐lived phosphorescence of Pdots, time‐gated luminescence imaging has been used to investigate the icO_2_, and the luminescence intensity images at different time range have been shown in Figure 4i–l. By collecting the photons at long lifetime ranges of 50–2000 ns and 125–2000 ns, and under different O_2_ levels, the images of cells under 2.5% O_2_ were brighter compared to those of cells under 21% O_2_, which confirmed that Pdots as a long‐lived icO_2_ probe was very beneficial for removing the short‐lived autofluorescence interference. Based on the above results, a ratiometric and time‐resolved phosphorescent/fluorescent probe platform has been constructed, which could be used to quantitatively detect icO_2_ levels and provide a more reliable and sensitive measurement by using PLIM and TGLI techniques.

### 2.7 Anticancer Investigation

**Figure 5:**
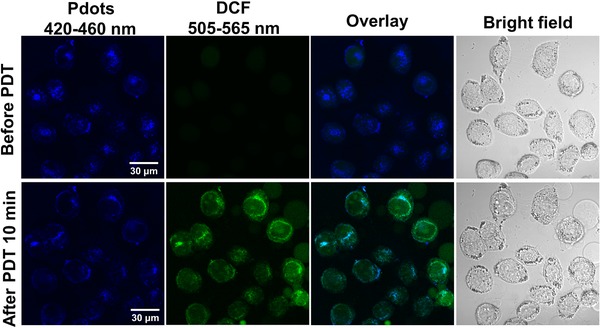
Subcellular localization of ROS generated during Pdots‐mediated PDT by DCFH‐DA staining. Cells were incubated with 20 μg mL^–1^ Pdots without irradiation (control cells) and irradiation of 532 nm light with 10 min. *λ*_ex_ = 405 nm.

As the good photosensitivity of porphyrin complexes for PDT,^18^ the potential applications of Pdots for PDT have been investigated. 1,3‐Diphenylisobenzofuran (DPBF) was used as an ^1^O_2_ indicator[[qv: 14e]] and hematoporphyrin (φ_∆_ = 0.65 in methanol)[[qv: 18c]] as a reference sensitizer to evaluate the ^1^O_2_ quantum yields of Pdots. Due to the lower photodamage effect and deeper tissue penetration of visible light than ultraviolet light for living cells,^19^ 532 nm laser was chosen as the PDT lamp. As shown in Figure S8 (Supporting Information), irradiation of the mixture of photosensitizer (Pdots or hematoporphyrin) and DPBF at 532 nm led to the decrease of the absorption band at 418 nm, indicating the generation of ^1^O_2_. The ^1^O_2_ quantum yield of Pdots was calculated to be 0.80 (Experimental Section). While in the control experiment (Figure S9a, Supporting Information), the absorption band at 418 nm was not changed. Such high photogeneration efficiency of singlet oxygen for Pdots will benefit for PDT application in cells, because ^1^O_2_ could induce the generation of reactive oxygen species (ROS) and trigger cell necrosis or apoptosis. To monitor the ^1^O_2_ generation of Pdots in living cells under light irradiation, 2,7‐dichlorifluorescein‐diacetate (DCFH‐DA) was used as a tracer agent, because it can be converted to DCFH in living cells, which is oxidized to fluorescent 2,7‐dichlorofluorescein (DCF) in the presence of ROS. The generation of ^1^O_2_ monitored by DCFH in aqueous solution was first investigated (Figure S10, Supporting Information). Irradiation of the mixture of phosphorescent Pdots and DCFH with a pulse light at 532 nm led to fluorescence turn on at 525 nm, indicating that the generation of ROS in PBS solutions can be monitored by DCFH. The unobservable fluorescence change in control experiment revealed that ^1^O_2_ generation was attributed to Pdots. Then, the intracellular ^1^O_2_ generation was monitored. After cells were coincubated with Pdots and DCFH‐DA, two emission channels of 420–460 and 505–565 nm were collected under different irradiation conditions, respectively (**Figure** **5**), which were attributed to the emission of Pdots and DCF. Increased intensity at emission channel of 505–565 nm was obtained after irradiation by 532 nm for 10 min. The emission intensity at 420–460 nm has no significant change after irradiation. Therefore, the generation of ROS in living cells was attributed to the Pdots under a pulse irradiation at 532 nm, which is proved in the control experiment (Figure S11, Supporting Information).

**Figure 6:**
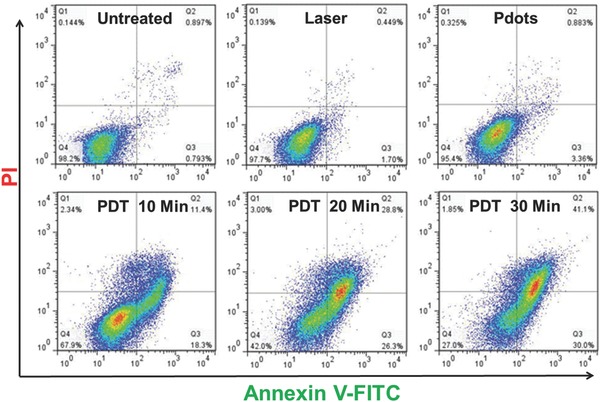
Flow cytometric analysis of cell death induced by Pdots‐mediated PDT.

**Figure 7:**
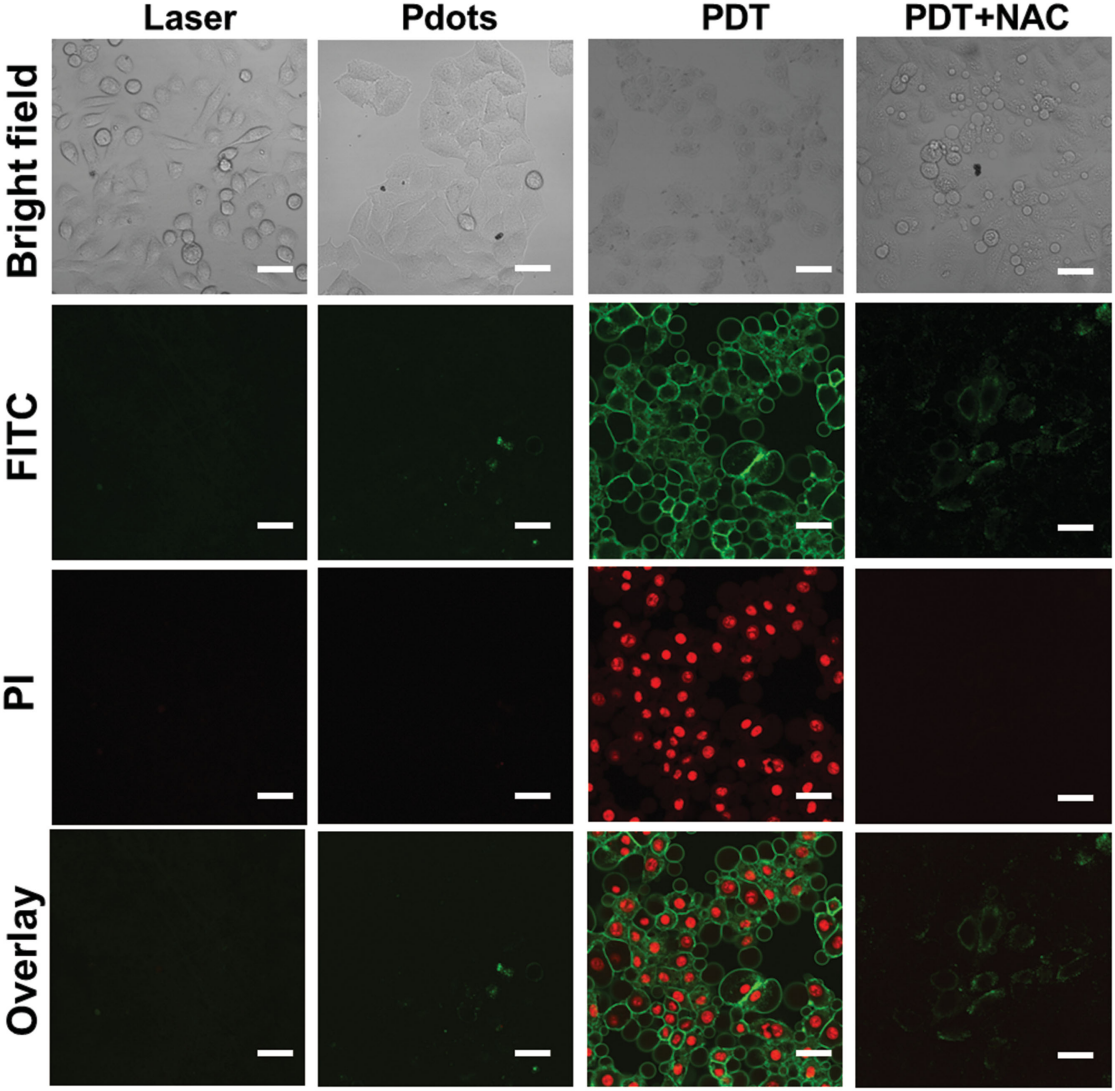
Confocal fluorescence images of Annexin V–FITC/PI stained HepG2 cells with different treatments. Scale bars: 40 μm. *λ*_ex_ = 488 nm.

**Figure 8:**
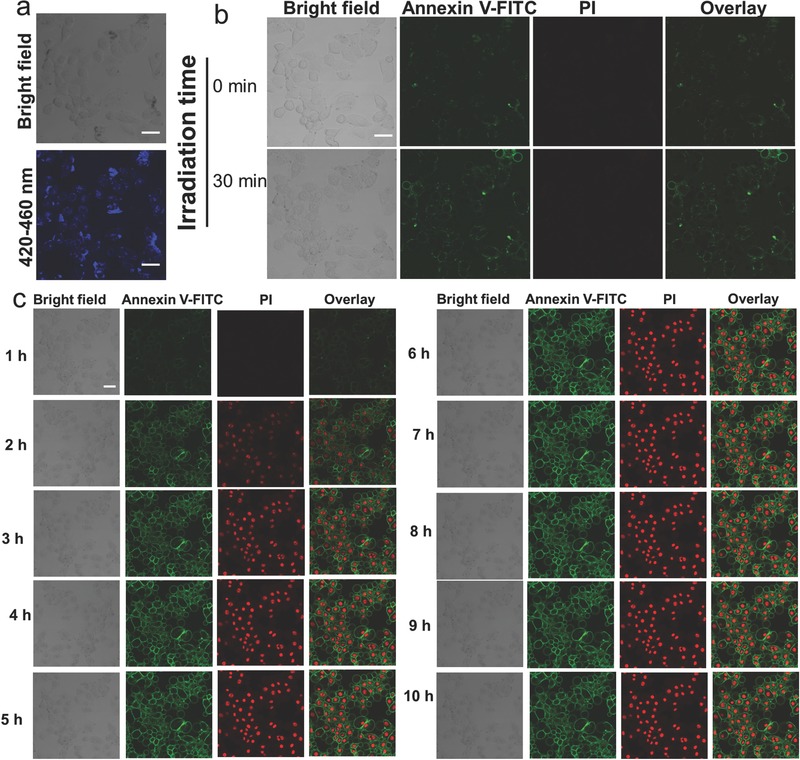
a) Photoluminescence images of Pdots loaded HepG2 cells. *λ*_ex_ = 405 nm. b) Photoluminescence images of Annexin V–FITC/PI stained Pdots loaded HepG2 cells with different irradiation times. c) Time‐lapse photoluminescence images of Annexin V–FITC/PI stained Pdots loaded HepG2 cells after 30 min light irradiation. *λ*_ex_ = 488 nm. Scale bars: 40 μm.

Additionally, the PDT effect of Pdots was studied using HepG2 cell line as a model. The cell death induced by Pdots‐mediated PDT was examined with the dual fluorescence of Annexin V–FITC/propidium iodide (PI), which was commonly used as the fluorescent probe to monitor and distinguish different stages of apoptosis cells. In flow cytometry assays, Annexin V–FITC^–^/PI^–^ (viable cells), Annexin V–FITC^+^/PI^–^ (early apoptotic cells), and Annexin V–FITC^+^/PI^+^ (necrotic or late‐stage apoptotic cells) determined the cells population at different stages of cell death. As shown in **Figure** **6**, an increased proportion of the apoptotic cells was obtained by prolonged irradiation time. This indicates the low dark cytotoxicity of Pdots and that an improved PDT effect was obtained by increasing the irradiation time. We tentatively consider that the high anticancer efficiency of Pdots can be attributed to the high efficient triplet–triplet energy transfer (TTET) from Pt(II) porphyrin complex to ground state oxygen. To ensure the effect of photoinduced ROS in the cancer cells, the real‐time fluorescence imaging was used to monitoring the progress of photoinduced cell apoptosis. After HepG2 cells were incubated with Pdots for 2 h and then irradiated by a 532 nm laser at a dose of 12 mW cm^–2^, cells were stained with Annexin V–FITC/PI and monitored for a long‐term observation via confocal laser scanning microscope. As shown in **Figure** **7**, a high efficiency of Pdots‐mediated PDT for killing cells was observed, while the irradiation‐induced cell apoptosis or death was prevented when Pdots‐loaded cells were coincubated with an ROS scavenger *N*‐acetyl‐l‐cysteine (NAC). Additionally, during the real‐time in situ observation of PDT induced cell death (**Figure** **8**), after irradiation of 532 nm light, increasing green fluorescence intensity of Annexin V–FITC was obtained, while no obvious red fluorescence of PI was observed (Figure 8b). These results indicated that the generated ROS in Pdots‐mediated PDT was responsible for the cell death. Further incubation of cells in dark, both the fluorescence signal of Annexin V–FITC and PI were detected after 2 h (Figure 8c), indicating that the cells were necrosis. However, in control experiment (Figures S12–S16, Supporting Information), efficient cell death was observed after 10 h later of Annexin V–FITC/PI staining, which was attributed to the lack of nutrients for too long time, leading to cell death at the end. Thus, the generated ROS of Pdots‐mediated PDT was responsible for the acceleration of cell death. To further confirm the good PDT performance of Pdots, the photoinduced cytotoxicity of Pdots in HepG2 cells was measured by using MTT assay (Figure S17, Supporting Information). The cell viability was reduced very quickly with the increase of irradiation time and dose of Pdots, which confirmed that the efficient PDT of Pdots for killing cancer cells. These results were consistent with the flow cytometry assay.

## 3 Conclusion

In summary, a water‐soluble and multifunctional phosphorescent Pdots has been designed and synthesized. The ratiometric imaging, PLIM and TGLI have been used to investigate the icO_2_ levels using this nanoprobe. The excellent performances of ratiometric imaging improved the accuracy of detection and the use of PLIM and TGLI could effectively remove the interference from autofluorescence to give more sensitive and reliable results. As the photosensitizer for PDT application, confirmed by MTT assay, flow cytometry analysis, and real‐time fluorescence imaging of photoinduced cell death in situ, the Pdots exhibited excellent ability to kill cancer cells, which is attributed to its high singlet oxygen quantum yield (0.80) by introducing oxygen‐sensitive phosphorescent Pt(II) porphyrin complex. These results demonstrated that the Pdots‐based diagnosis–therapy integrative systems show great potential in future biomedical applications.

## 4 Experimental Section

*Instruments and Methods*: NMR spectra were recorded on a Bruker Ultra Shield Plus 400 MHz NMR instrument (^1^H: 400 MHz, ^13^C: 125 MHz). Mass spectra were performed with a Bruker autoflex MALDI‐TOF/TOF mass spectrometer. The UV–visible absorption spectra were obtained with a Shimadzu UV‐3600 UV–vis–NIR spectrophotometer. Photoluminescent spectra were measured using an RF‐5301PC spectrofluorophotometer. Lifetime studies were performed with an Edinburgh FL920 photocounting system with a semiconductor laser as the excitation source. TEM was conducted on a JEOL JEM‐2100 transmission electron microscope at an acceleration voltage of 100 kV. The number‐average molecular weight (*M*_n_) and weight‐average molecular weight (*M*_w_) of the polymers were characterized in THF by gel permeation chromatography at 35 °C (polystyrene as standard). The actual contents of different monomers in the polymer were determined by ^1^H NMR (in CDCl_3_). For oxygen sensing, standard gas mixtures containing 0%, 2%, 4%, 10%, 12%, 16%, 18%, and 21% of O_2_ balanced with N_2_ (100%, 98%, 96%, 90%, 88%, 84%, 82%, and 79%, respectively) were passed through the cuvette for 10 min to equilibrate the oxygen content to the respective concentrations and to monitor the changes of polymer luminescence.

*Materials*: All reagents, unless specified, were purchased from Sigma‐Aldrich, Acros, and Alfa, and used without further purification. All solvents were purified before use. All reactions were performed in a nitrogen atmosphere.

*Synthesis of 5‐(Pentafluorophenyl)dipyrromethane*:^20^ Pyrrole (25 mL, 360 mmol) and 2,3,4,5,6‐pentafluorobenzaldehyde (5.34 g, 27.2 mmol) were added to a 100 mL round‐bottomed flask. The solution was degassed with a stream of nitrogen for 20 min. Trifluoroacetic acid (210 μL, 2.72 mmol) was added, and the mixture was stirred under nitrogen and in dark at room temperature for 1.5 h. The mixture turned yellow during the reaction. NaOH (0.1 m, 20 mL) was added to quench the reaction. After stirring for 3 h to make sure that the reaction was quenched completely, a blue mixture was obtained. Ethyl acetate (100 mL) was then added. The organic phase was washed with 4 × 80 mL of water and then dried by anhydrous Na_2_SO_4_. The crude product obtained after removal of solvents was purified by column chromatography [silica, 6 cm dia. × 14 cm, hexanes/CH_2_Cl_2_/ethyl acetate (10:2:1)] to give a white solid (6.56 g, 77% yield). ^1^H NMR (400 MHz, CDCl_3_): *δ* (ppm) = 8.16 (br s, 2H), 6.73 (td, *J* = 2.8 Hz, 1.6 Hz, 2H), 6.17 (dd, *J* = 6 Hz, 2.8 Hz, 2H), 6.06–6.01 (m, 2H), 5.90 (s, 1H).

*Synthesis of 5,15‐Bis(pentafluorophenyl)‐10,20‐bis(4‐bromophenyl)porphyrin*: 4‐Bromobenzaldehyde (0.583 g, 3.2 mmol) and 5‐(pentafluorophenyl)dipyrromethane (1 g, 3.2 mmol) were added to 320 mL of deaerated CH_2_Cl_2_ in a dry round‐bottomed 500 mL flask and the solution was degassed with a stream of Ar for 10 min. BF_3_·O(Et)_2_ (0.278 mL) was then added, and the solution was stirred under Ar at room temperature for 1 h, and then 2,3‐dichloro‐5,6‐dicyano‐1,4‐benzoquinone (DDQ; 572 mg 2.5 mmol) was added. The mixture was stirred at room temperature for an additional 1 h, and then the solvent was removed. The pure product was obtained by column chromatography (silica, 1/10 CH_2_Cl_2_/hexane v/v) (0.9 g, 20% yield). ^1^H NMR (400 MHz, CDCl_3_): *δ* (ppm) = 8.94 (d, *J* = 4.8 Hz, 2H), 8.87 (d, *J* = 5.6 Hz, 4H), 8.80 (d, *J* = 4.8 Hz, 2H), 8.05–8.10 (m, 4H), 7.90–7.96 (m, 4H), −2.83 (br s, 2H). MS: calcd. for C_44_H_18_N_4_F_10_Br_2_ 952.43, found: 953.07.

*Synthesis of Platinum(II) 5,15‐Bis(pentafluorophenyl)‐10,20‐bis(4‐bromophenyl)porphyrin (M_1_)*: K_2_[PtCl_4_] (93.7 mg, 0.226 mmol) and 5,15‐bis(pentafluorophenyl)‐10,20‐bis(4‐bromophenyl)‐porphyrin (71.7 mg, 0.0753 mmol) were added to a dry round‐bottomed 25 mL flask with 7 mL of anhydrous benzonitrile and degassed with a stream of Ar for 10 min. The solution was stirred and refluxed under Ar at 180 °C for 2 d, the mixture was cooled, and then the solvent was removed under vacuum. The crude product was purified with column chromatography on silica gel with CH_2_Cl_2_ as the eluent. Recrystallization from CH_2_Cl_2_/hexane gave 30 mg of Pt(II) porphyrin complex as brown‐red crystals (35% yield). ^1^H NMR (400 MHz, CDCl_3_): *δ* (ppm) = 8.84 (d, *J* = 5.2 Hz, 4H), 8.73 (d, *J* = 5.2 Hz, 4H), 8.00–8.07 (m, 4H), 7.88–7.95 (m, 4H). MS: calcd. for C_44_H_16_N_4_F_10_Br_2_Pt 1145.34, found: 1146.37.

*Synthesis of Polymer 1 (P1)*: The monomers (M_3_ and M_4_) were synthesized according to the previous reports.^21^ A mixture of M_1_ (30 mg, 0.025 mmol), M_2_ (4 mg, 0.0125 mmol), M_3_ (83 mg, 0.130 mmol), M_4_ (63 mg, 0.085 mmol), [Pd(PPh_3_)_4_] (5 mg), and tetrabutylammoniumbromide (TBAB) was placed in 5 mL of toluene and 3.7 mL of 2.0 m potassium carbonate aqueous solution, which was degassed. The mixture was vigorously stirred at 85 °C under a nitrogen atmosphere for 2 d. After cooling down to room temperature, water (20 mL) was added and the mixture was extracted with CH_2_Cl_2_. The organic layer was dried over anhydrous MgSO_4_ and the solvent was removed. The crude product was dissolved in THF (0.5 mL) and then precipitated in CH_3_OH/H_2_O (10:1). The precipitate was collected by filtration, and dissolved in THF again and reprecipitated in methanol (200 mL). The precipitate was filtered and washed with acetone and then dried in vacuum for 24 h to afford the desired polymer (80 mg, 45% yield) as a red solid. ^1^H NMR (400 MHz, CDCl_3_): *δ* (ppm) = 8.60–9.12 (m, 0.84H pyrrole H of porphyrin), 8.06–8.43 (m, 0.84H Ar H of porphyrin), 7.45–7.98 (m, 8H, Ar H of fluorene and benzene), 3.30 (t, 4H CH_2_Br of fluorene), 2.16 (br, 4H CH_2_ of fluorene), 1.70 (br, 4H CH_2_ of fluorene), 1.0–1.5 (m, 8H CH_2_ of fluorene), 0.72–0.97 (m, 4H CH_2_ of fluorene 4H). ^13^C NMR (125 Hz, CDCl_3_): 151.51, 140.52, 140.12, 127.23, 127.21, 126.33, 121.34, 120.18, 55.34, 40.32, 34.04, 32.63, 29.09, 27.73, 23.72. GPC (THF, polystyrene standard), *M*_w_: 81 490 g mol^−1^; *M*_n_: 65 490 g mol^−1^; PDI: 1.25.

*Synthesis of Polymer 2 (P2)*: Condensed trimethylamine (2 mL) was added dropwise to a solution of P1 (40 mg) in THF (10 mL) at −78 °C using a dry ice–acetone bath. The mixture was allowed to warm up to room temperature. The precipitate was redissolved by the addition of methanol (10 mL). After the mixture was cooled down to −78 °C, extra trimethylamine (2 mL) was added and the mixture was stirred for 24 h at room temperature. After removal of most of the solvent, acetone was added to precipitate P2 (35 mg, 72% yield) as the red powder. ^1^H NMR (400 MHz, CDCl_3_): *δ* (ppm) = 8.50–9.12 (m, 0.84H), 8.06–8.43 (m, 0.84H), 7.50–7.95 (m, 8H), 3.30 (t, 4H), 3.1 (s, 16H), 2.3 (br, 4H), 1.6 (br, 4H), 1.3 (br, 8H), 0.8 (br, 4H). ^13^C NMR (125 MHz, CD_3_OD): *δ* 151.8, 140.9, 140.4, 140.0, 127.6, 126.1, 121.2, 120.5, 66.7, 55.7, 52.5, 40.2, 29.2, 25.8, 23.7, 22.5. Quarternization degree: 90%.

*Polymer Dots*: 1.2 mg P2 was dissolved in 5.0 mL methanol by stirring overnight under inert atmosphere. First, 2 mL of P2 solutions was injected quickly into 8 mL PBS while sonicating the mixture to aid mixing. Then, the resulting nanoparticle suspension was filtered through a 0.2 μm membrane filter in order to remove lager aggregates. The methanol was removed by evaporation under vacuum at 38 °C. Additional filtration step was carried out to acquire Pdots.

*Cell Culture and Imaging*: Human hepatoma cells HepG2 were cultured in RPMI 1640 (Dulbecco's modified Eagle's medium) (Invitrogen), containing 10% fetal bovine albumin (Invitrogen) and 1% penicillin streptomycin (Invitrogen). Cells were maintained at 37 °C under an atmosphere of 5% CO_2_ in air.

Confocal luminescence imaging was carried out on an Olympus IX81 laser scanning confocal microscope equipped with a 40 immersion objective lens. A semiconductor laser was served as excitation of the HepG2 cells incubated with Pdots probes at 405 nm. The emission was collected at 420–460 and 630–680 nm, respectively, for the HepG2 cells incubated with Pdots. Pdots were added to RPMI 1640 to yield 7.5 μg mL^−1^ solution. The HepG2 cells were incubated with the Pdots for 2 h at 37 °C.

The PLIM image setup is integrated with Olympus IX81 laser scanning confocal microscope. The photoluminescence signal was detected by the system of the confocal microscope and correlative calculation of the data was performed by professional software which was provided by PicoQuant Company. The light from the pulse diode laser head (PicoQuant, PDL 800‐D) with excitation wavelength of 405 nm and frequency of 0.5 MHz was focused onto the sample with a 40×/NA 0.95 objective lens for single‐photon excitation.

Phase contrast bright‐field and fluorescence images were taken on a fluorescence microscope (Olympus 1 × 71) with a mercury lamp (200 W) as light source. Experiments for light‐induced anticancer activity of cancer cells were performed with diode pumped solid‐state laser that simulated 532 nm light source. The intensities of incident beams were checked by a power and energy meter.

*Singlet Oxygen Quantum Yields*: The quantum yield for ^1^O_2_ photosensitization has been measured according to the reported method[[qv: 13e]] using DPBF as an ^1^O_2_ indicator and hematoporphyrin (φ_∆_ = 0.65) as a standard in methanol. The experiment was conducted for Pdots (12 μg mL^−1^) in 10 × 10^−3^ m PBS solution containing DPBF (5 × 10^−6^ m) and hematoporphyrin (2.5 × 10^−6^ m) in methanol containing DPBF, respectively. The absorption spectra were recorded at 15 min intervals under irradiation at 532 nm laser (2 mW cm^−2^). DPBF oxidation was monitored by UV–vis–NIR spectrophotometer. The ^1^O_2_ quantum yield was calculated by monitoring the absorption spectral change at 418 nm of DPBF with the following equation

(2) $$\Phi_{\Delta (\mathrm{Pdots})}=\Phi_{\Delta (\mathrm{std})}(\kappa_{\mathrm{Pdots}}/\kappa_{\mathrm{std}})(F_{\mathrm{std}}/F_{\mathrm{Pdots}})$$

where subscripts std designate the hematoporphyrin, *κ* stands for the slope of plot of the absorbance of DPBF (at 418 nm) versus irradiation time. *F* stands for the absorption correction factor, which is given by *F* = 1 ‐ 10^−OD^ (OD represents the optical density of Pdots sample and hematoporphyrin at 532 nm).

*ROS Measurements In Vitro*:[[qv: 13b]] The DCFH‐DA was converted to DCFH by 0.01 × 10^−3^ m NaOH. The final concentration of DCFH was 40 × 10^−3^ m. To 1.0 mL of the activated DCFH solution were added compounds. The fluorescence spectra were measured after the specimens were irradiated with 532 nm laser (2 mW cm^−2^). Fluorescence spectra of DCF solution were recorded in 500–700 nm emission range with the excitation wavelength of 488 nm.

*ROS Measurements in Intracellular*: After the HepG2 cells were incubated with Pdots for 2 h, they were further incubated with 10 × 10−^6^ m DCFH‐DA for 20 min and irradiated with a 532 nm laser at a power of 12 mW cm^–2^ for 0 and 10 min, to perform the fluorescence detection of DCF with the CLSM, respectively, which could give the level of intracellular ROS. The emission was collected at 420–460 and 505–565 nm, respectively.

*Assay for Cell Viability Population by Flow Cytometry*: The HepG2 cells were seeded in the six‐well plates at a density of ≈1 × 10^5^ for 24 h, 37 °C. Then the cells were washed once with PBS. RPMI 1640 medium (2 mL) with 40 μg phosphorescent Pdots was added for 2 h further incubation. The medium was then replaced with fresh culture medium and irradiated by 532 nm laser at a power of 12 mW cm^–2^ for 0, 10, 20, and 30 min, respectively. Afterward, the cells were stained with Annexin V–FITC/PI according to the manufacturer's instruction, trypsinized, harvested, rinsed with PBS, resuspended, and subjected to perform flow cytometric assay using BD FACSCanto II flow cytometry.

*Assay for Cell Viability by MTT*: HepG2 cells were incubated in RPMI 1640 (high glucose) medium containing 10% FBS. All cells were harvested and subcultured in 96‐well plates at a density of 4 × 10^4^ cells/well for 24 h in a humidified atmosphere containing 5% CO_2_ at 37 °C. Pdots with varying concentrations were added into the wells, respectively, and further cultured for another 2 h. After that, the culture media containing polymers were discarded with fresh cell growth medium (200 μL). The 532 nm light with a dose of 12 mW cm^−2^ was used to perform PDT treatment. After irradiation, the cells were allowed to continue growing for 24 h. Then MTT (5 mg mL^−1^, 10 μL/well) was added to the wells and the cells were incubated at 37 °C for another 4 h. DMSO (200 μL/well) was added to dissolve the produced formazan after discarding the supernatant. The plates were shaken for 10 min and the absorbance values of the wells were then read with microplate reader at 570 nm. The cell viability rate (VR) was calculated according to the following equation: VR = (*A*_experimental group_/*A*_control group_) × 100%.

*Confocal Imaging of Photoinduced Cell Death*: The HepG2 cells were seeded in the culture plates (Nunc) at 37 °C for 24 h, and then the cells were washed once with PBS. RPMI 1640 medium (2 mL) with 40 μg phosphorescent Pdots was added. After the cells were further cultured for 2 h, the cells were stained with Annexin V–FITC/PI, the plates were irradiated under 532 nm light for 30 min, and the cell death imaging was visualized with CLSM at time‐lapse model. Collecting ranges were 505–565 nm for Annexin V–FITC emission and 600–700 nm for PI emission, respectively.

In the presence of NAC, the irradiation‐induced cell death was also examined by CLSM. After the Pdots loaded cells were incubated with 2.5 × 10^–3^ m NAC for 30 min, the plates were irradiated under 532 nm light for 30 min and then the cells were stained with Annexin V–FITC/PI to visualize the cell death with CLSM at time‐lapse model.

## Supporting information

As a service to our authors and readers, this journal provides supporting information supplied by the authors. Such materials are peer reviewed and may be re‐organized for online delivery, but are not copy‐edited or typeset. Technical support issues arising from supporting information (other than missing files) should be addressed to the authors.

Supplementary

## References

[1] a) M. D. Brand , D. G. Nicholls , Biochem. J. 2011, 435, 297; 21726199 10.1042/BJ20110162 PMC3076726

[2] a) W. R. Wilson , M. P. Hay , Nat. Rev. Cancer 2011, 11, 393; 21606941 10.1038/nrc3064

[3] A. L. Burgueno , T. F. Gianotti , N. G. Mansilla , C. J. Pirola , S. Sookoian , Clin. Sci. 2013, 124, 53. 22827449 10.1042/CS20120151

[4] Y. Y. Fan , W. W. Hu , H. B. Dai , J. X. Zhang , L. Y. Zhang , P. He , Y. Shen , H. Ohtsu , E. Q. Wei , Z. Chen , J. Cereb. Blood Flow Metab. 2011, 31, 305. 20588322 10.1038/jcbfm.2010.94 PMC3049494

[5] B. V. Zlokovic , Nat. Rev. Neurosci. 2011, 12, 723. 22048062 10.1038/nrn3114 PMC4036520

[6] a) M. C. Krishna , S. English , K. Yamada , J. Yoo , R. Murugesan , N. Devasahayam , J. A. Cook , K. Golman , J. H. Ardenkjaer‐Larsen , S. Subramanian , J. B. Mitchell , Proc. Natl. Acad. Sci. USA 2002, 99, 2216; 11854518 10.1073/pnas.042671399 PMC122345

[7] a) R. Timmerman , R. McGarry , C. Yiannoutsos , L. Papiez , K. Tudor , J. DeLuca , M. Ewing , R. Abdulrahman , C. DesRosiers , M. Williams , J. Fletcher , J. Clin. Oncol. 2006, 24, 4833; 17050868 10.1200/JCO.2006.07.5937

[8] a) A. Fercher , S. M. Borisov , A. V. Zhdanov , I. Klimant , D. B. Papkovsky , ACS Nano 2011, 5, 5499; 21671589 10.1021/nn200807g

[9] a) D. B. Papkovsky , R. I. Dmitriev , Chem. Soc. Rev. 2013, 42, 8700; 23775387 10.1039/c3cs60131e

[10] a) S. Takahashi , W. Piao , Y. Matsumura , T. Komatsu , T. Ueno , T. Terai , T. Kamachi , M. Kohno , T. Nagano , K. Hanaoka , J. Am. Chem. Soc. 2012, 134, 19588; 23157219 10.1021/ja310049d

[11] a) T. Yoshihara , Y. Yamaguchi , M. Hosaka , T. Takeuchi , S. Tobita , Angew. Chem., Int. Ed. 2012, 51, 4148; 10.1002/anie.201107557 22344795

[12] a) H. Liu , H. Yang , X. Hao , H. J. Xu , Y. Lv , D. B. Xiao , H. D. Wang , Z. Y. Tian , Small 2013, 9, 2639; 23519925 10.1002/smll.201203127

[13] a) H. B. Sun , S. J. Liu , W. P. Lin , K. Y. Zhang , W. Lv , X. Huang , F. W. Huo , H. R. Yang , G. Jenkins , Q. Zhao , W. Huang , Nat. Commun. 2014, 5, 3601; 24710282 10.1038/ncomms4601

[14] a) C. F. Xing , G. M. Yang , L. B. Liu , Q. Yang , F. T. Lv , S. Wang , Small 2012, 8, 524; 22223534 10.1002/smll.201101825

[15] a) S. P. Li , C. T. Lau , M. W. Louie , Y. W. Lam , S. H. Cheng , K. K. Lo , Biomaterials 2013, 34, 7519; 23849346 10.1016/j.biomaterials.2013.06.028

[16] a) Q. Zhu , F. Qiu , B. S. Zhu , X. Y. Zhu , RSC Adv. 2013, 3, 2071;

[17] a) C. F. Wu , T. Schneider , M. Zeigler , J. B. Yu , P. G. Schiro , D. R. Burnham , J. D. McNeill , D. T. Chiu , J. Am. Chem. Soc. 2010, 132, 15410; 20929226 10.1021/ja107196s PMC2965818

[18] a) B. Zhao , J. J. Yin , P. J. Bilski , C. F. Chignell , J. E. Roberts , Y. Y. He , Toxicol. Appl. Pharmacol. 2009, 241, 163; 19695274 10.1016/j.taap.2009.08.010 PMC2783992

[19] a) A. R. Lehmann , S. K. Bell , C. F. Arlett , S. A. Harcourt , E. A. Weerd‐Kastelein , W. Keijzer , P. H. Smith , Cancer Res. 1977, 37, 904; 837385

[20] J. K. Laha , S. Dhanalekshmi , M. Taniguchi , A. Ambroise , J. S. Lindsey , Org. Process Res. Dev. 2003, 7, 799.

[21] a) H. F. Shi , X. J. Chen , S. J. Liu , H. Xu , Z. F. An , L. Ouyang , Z. Z. Tu , Q. Zhao , Q. L. Fan , L. H. Wang , W. Huang , ACS Appl. Mater. Interfaces 2013, 5, 4562; 23527622 10.1021/am4000408

