# Supplementary material for: Multifunctional Phosphorescent Conjugated Polymer Dots for Hypoxia Imaging and Photodynamic Therapy of Cancer Cells
Source: Adv Sci (Weinh). 2015 Sep 10;3(2):1500155. doi: 10.1002/advs.201500155 (PMC5049659; doi:10.1002/advs.201500155)
Supplement: Supplementary file 1 — Supplementary [file ADVS-3-0a-s001.pdf]

Copyright WILEY-VCH Verlag GmbH & Co. KGaA, 69469 Weinheim, Germany, 2015.

ADVANCED SCIENCE
Open Access

Supporting Information

for *Adv. Sci.*, DOI: 10.1002/advs. 201500155

Multifunctional Phosphorescent Conjugated Polymer Dots for Hypoxia Imaging and Photodynamic Therapy of Cancer Cells

Xiaobo Zhou, Hua Liang, Pengfei Jiang, Kenneth Yin Zhang, Shujuan Liu, Tianshe Yang, Qiang Zhao, Lijuan Yang, Wen Lv, Qi Yu, and Wei Huang**

Copyright WILEY-VCH Verlag GmbH & Co. KGaA, 69469 Weinheim, Germany, 2015.

Supporting Information

Multifunctional Phosphorescent Conjugated Polymer Dots for Hypoxia Imaging and Photodynamic Therapy of Cancer Cells

Xiaobo Zhou, Hua Liang, Pengfei Jiang, Kenneth Yin Zhang, Shujuan Liu, Tianshe Yang, Qiang Zhao, Lijuan Yang, Wen Lv, Qi Yu, and Wei Huang**

X. B. Zhou, H. Liang, P. F. Jiang, Dr. K. Y. Zhang, Prof. S. J. Liu, Dr. T. S. Yang, Prof. Q. Zhao, L. J. Yang, W. Lv, Q. Yu, Prof. W. Huang
Key Laboratory for Organic Electronics and Information Displays & Institute of Advanced Materials (IAM)
Jiangsu National Synergetic Innovation Center for Advanced Materials (SICAM)
Nanjing University of Posts & Telecommunications (NUPT)
Nanjing 210023, Jiangsu, China.
E-mail: iamqzhao@njupt.edu.cn
Prof. W. Huang
Key Laboratory of Flexible Electronics (KLOFE) & Institute of Advanced Materials (IAM)
Jiangsu National Synergetic Innovation Center for Advanced Materials (SICAM)
Nanjing Tech University (NanjingTech)
Nanjing 211816, Jiangsu, China.
E-mail: wei-huang@njtech.edu.cn

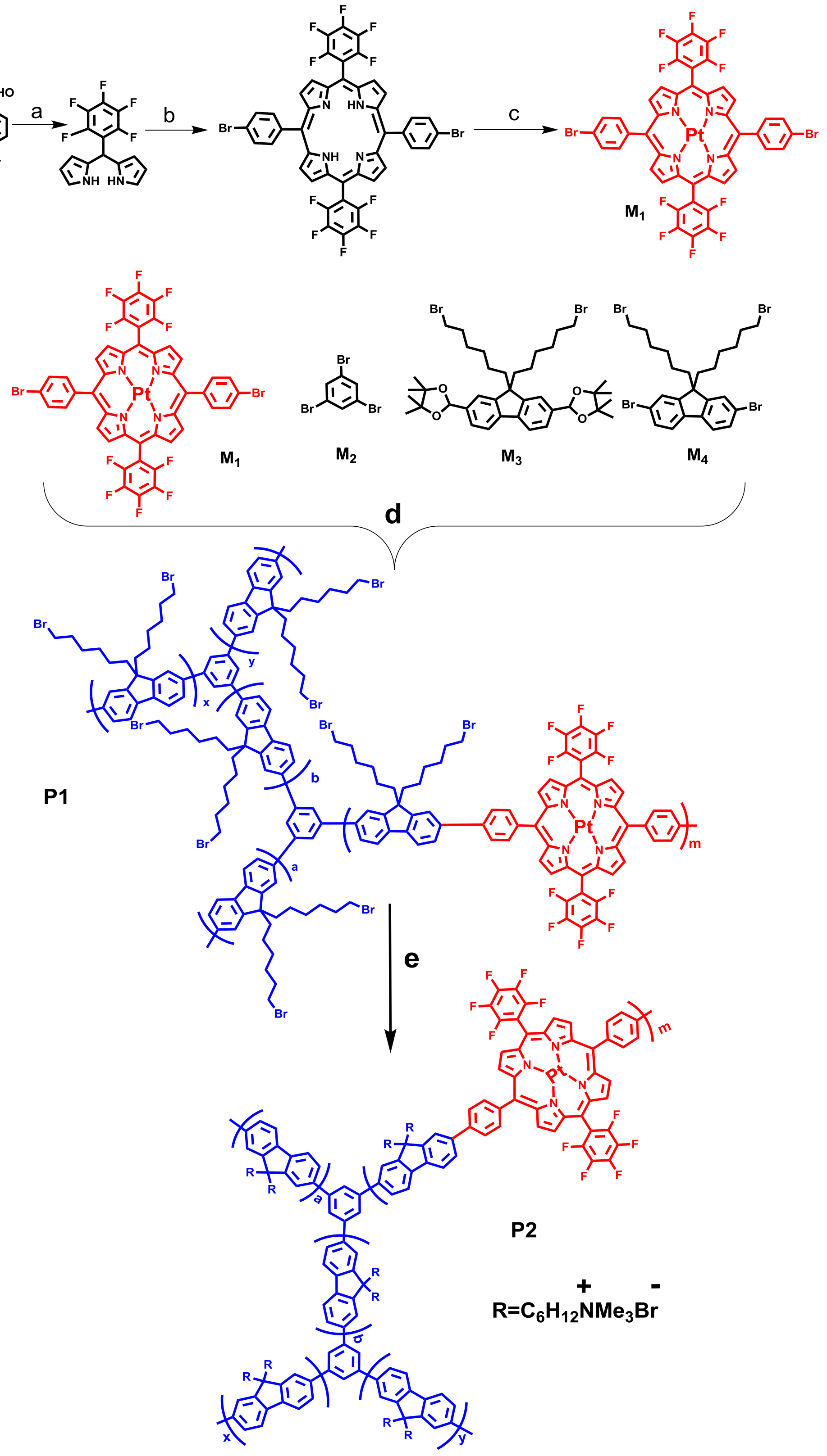

Scheme S1. The synthetic route of **P2**. (a) i. Pyrrole, trifluoroacetic acid, rt, 1.5 h, ii. NaOH, 3 h, 77%; (b) i. 4-Bromobenzaldehyde, $BF_3{\cdot}O(Et)_2$, CH_2Cl_2, rt, 1 h, ii. DDQ, rt, 1 h, 20%; (c) $K_2[PtCl_4]$, benzonitrile, 180 °C, 48 h, 35%; (d) $[Pd(PPh_3)_4]$, tetrabutylammoniumbromide (TBAB), toluene, K_2CO_3, 80 °C, 72 h, 80%; (e) Trimethylamine, THF, 48 h, 72%.

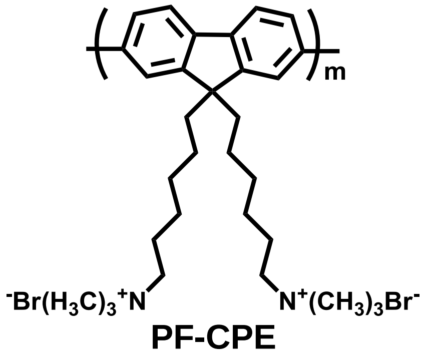

Figure S1. The chemical structure of PF-CPE.

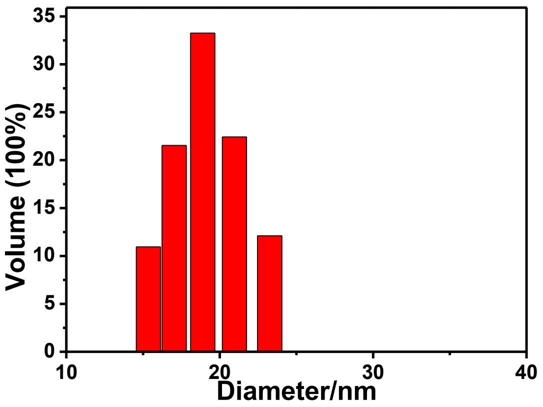

Figure S2. Dynamic light scattering of phosphorescent Pdots in aqueous solution.

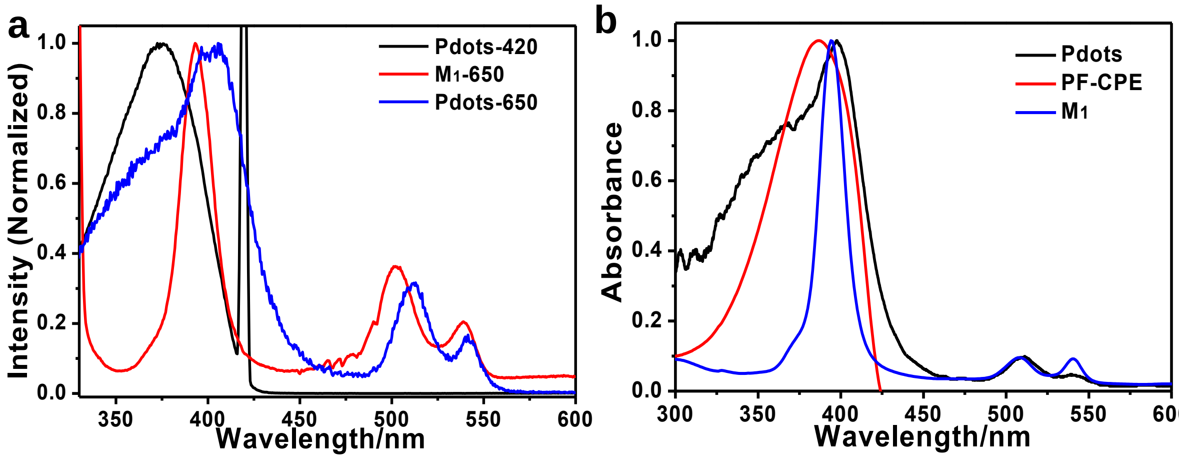

Figure S3. a) Excited spectra of M_1 in CH_2Cl_2, Pdots in aqueous at 650 nm and 420 nm. The soret band at 420 nm in the excited spectra of Pdots-420 was attributed to the emission of Pdots. b) Absorption spectra of Pdots and PF-CPE in aqueous solutions and M_1 in CH_2Cl_2.

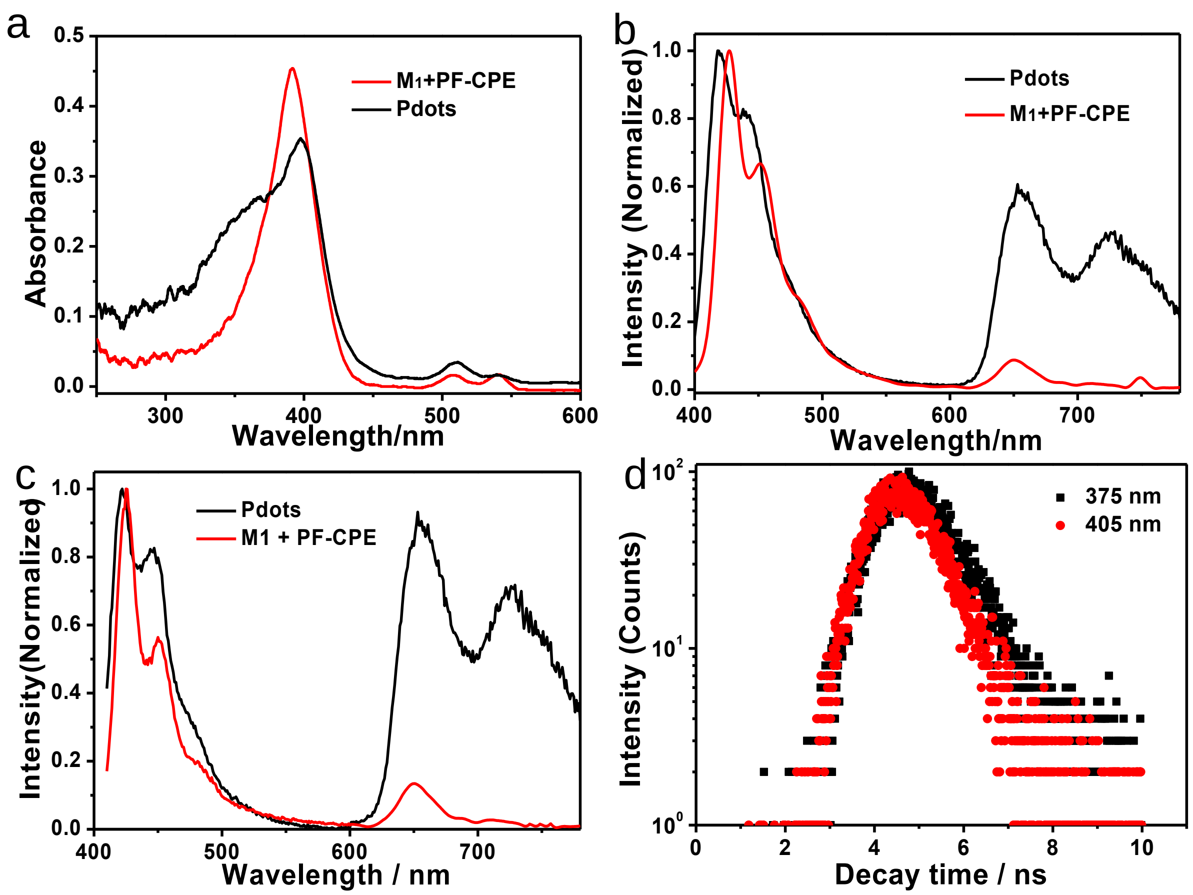

Figure S4. a) Absorption spectra, b) 375 nm excited and c) 405 nm excited emission spectra of Pdots and the blend of 2.1 μg/mL (1.8 μM) Pt(II) porphyrin (**M_1**) and 10.0 μg/mL (16.2 μM, equvilent concerntration of fluorene units) Pt(II)-free polyfluorene PF-CPE in aqueous solution. The molar ratio of Pt: fluorene was calculated as 10 : 90; d) Luminescence decays at 420 nm of Pdots which were excited by 405 nm and 375 nm light in aqueous solution, respectively. Excitation lamp: hydrogen light.

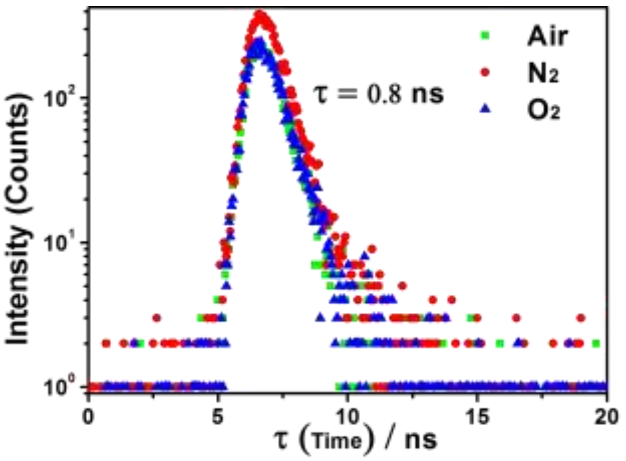

Figure S5. Luminescence decays of Pdots at 420 nm in aqueous solution saturated with N_2, air and O_2, respectively**.** The temporal resolution for the transient photoluminescence experiments is 200 ps.

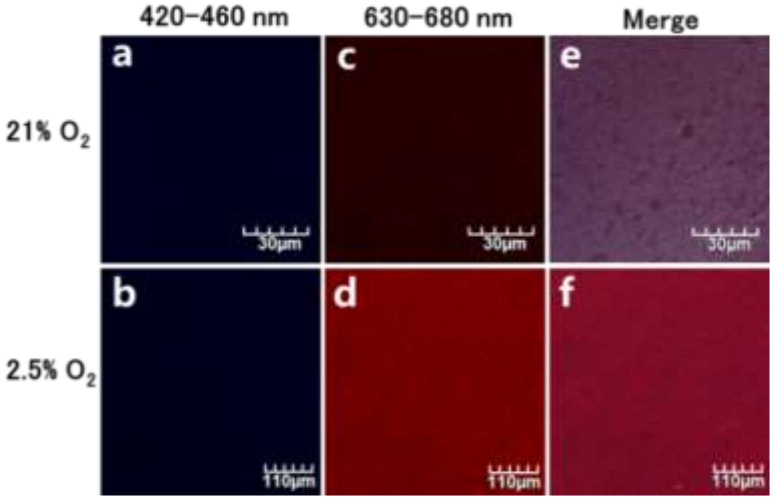

Figure S6. Photoluminescence images of Pdots film under air (top) and 2.5% O_2 (bottom). Fluorescent emission of polyfluorene at 420-460 nm (a, b), phosphorescent emission of 5, 15-*bis* (pentafluorophenyl)-10,20-*bis*(phenyl) platinum(II) porphyrin at 630-680 nm (c, d), and the overlay of brightfield image and luminescent images collected at 420-460 nm and 630-680 nm (e, f). λ_{ex} = 405 nm.

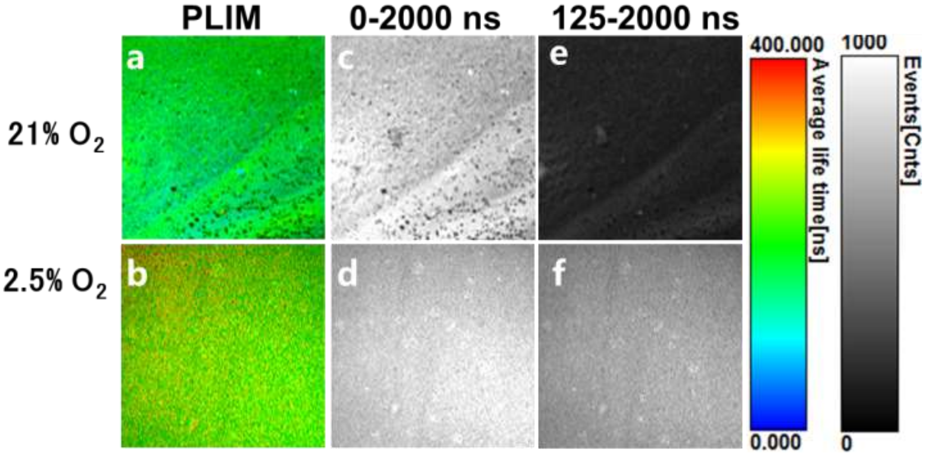

Figure S7. Photoluminescence lifetime images (a, b) and time-gated luminescence images with different delay time (c-f) of Pdots films under air (top) and 2.5% O_2 (bottom). λ_{ex} = 405 nm.

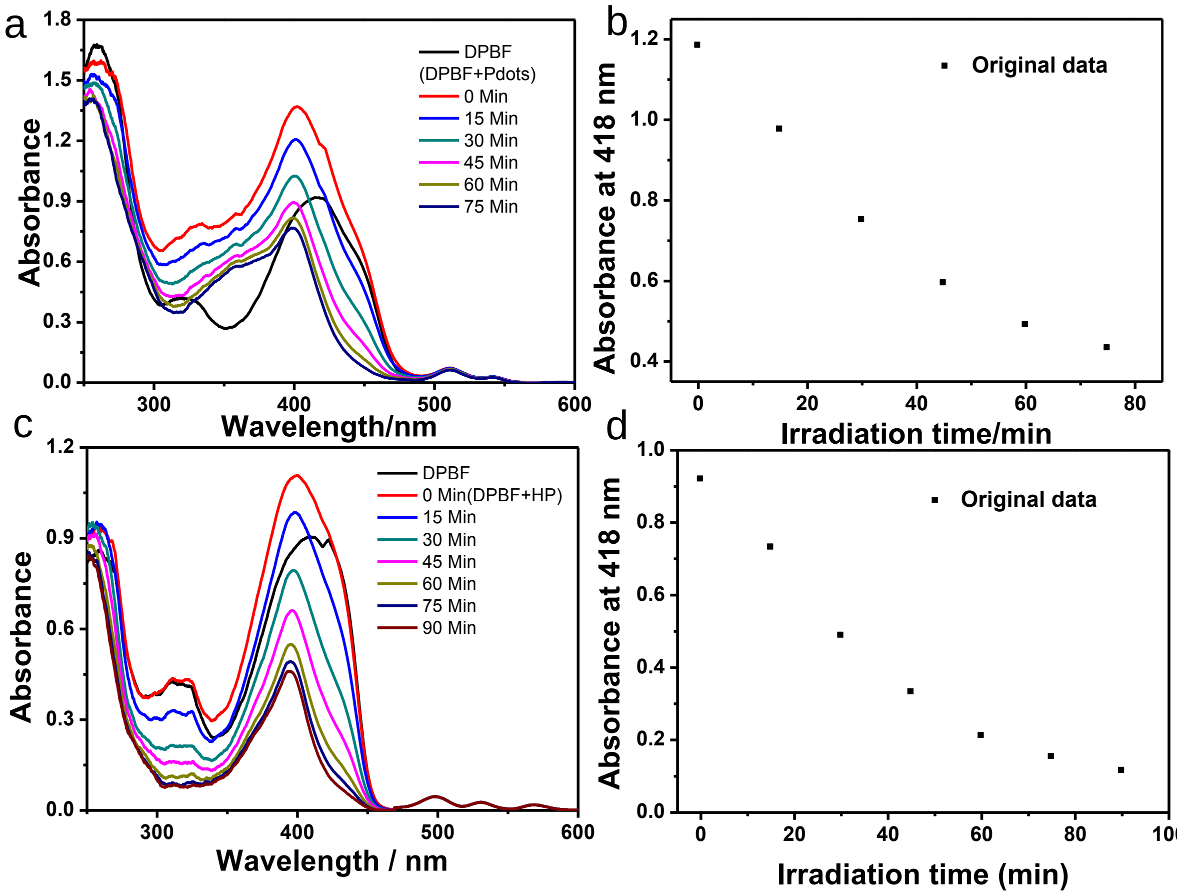

Figure S8. Absorption spectra of single oxygen trap DPBF (40 μM) with (a) Pdots (12 μg) in 10 mM PBS buffer and (c) hematoporphyrin (2.5 μM) in methanol under irradiation at 532 nm for different irradiation time (0–75 min); plot of the absorbance at 418 nm as a function of light irradiation time in a (b) PBS buffer solution containing Pdots and DPBF and (d) methanol containing hematoporphyrin and DPBF.

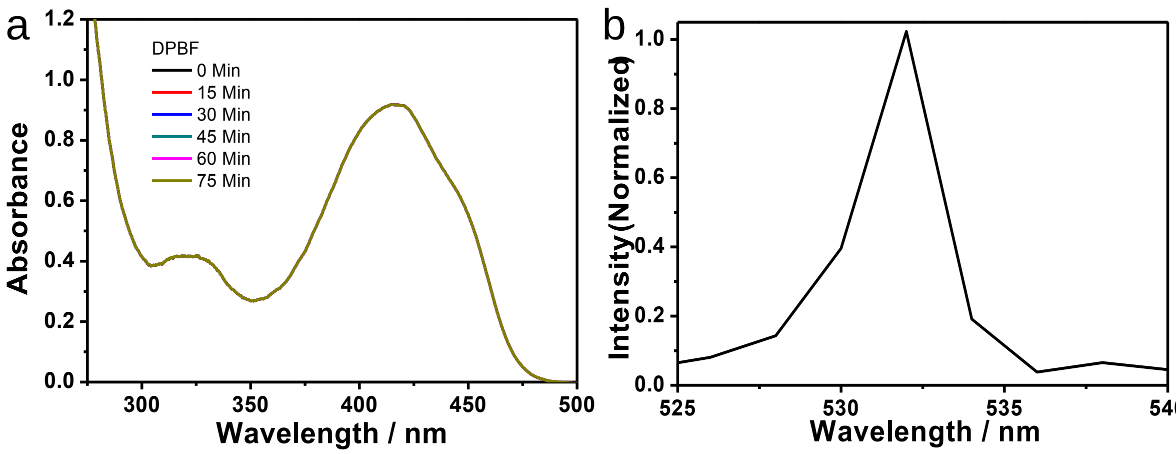

Figure S9. a) Absorption spectra of single oxygen trap DPBF (40 μM) alone in 10 mM PBS buffer under irradiation at 532 nm for different time (0–75 min); b) Spectral profile of the LED array lamp used for cell irradiation.

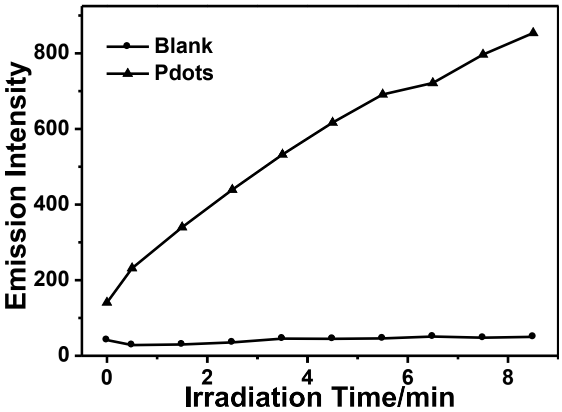

Figure S10. DCF emission intensity at 525 nm as a function of 532 nm light irradiation time in aqueous solution in the presence of Pdots (12 μg/mL) and without polymer. λ_{ex} = 488 nm.

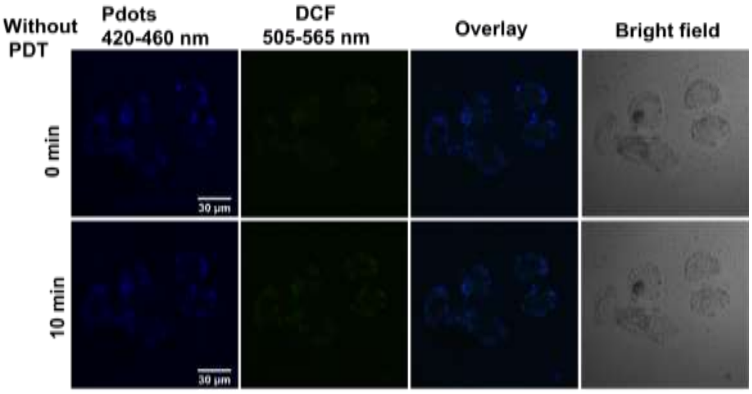

Figure S11. Subcellular localization of ROS generated during incubation of Pdots without irradiation a) and irradiation of 532 nm light without incubation of Pdots by DCFH-DA staining.

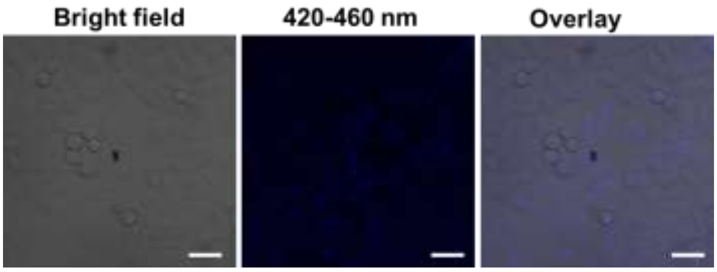

Figure S12. Photoluminescence images of HepG2 cells co-incubated with Pdots and NAC before light irradiation. Scale bars: 40 μm. λ_{ex} = 405 nm.

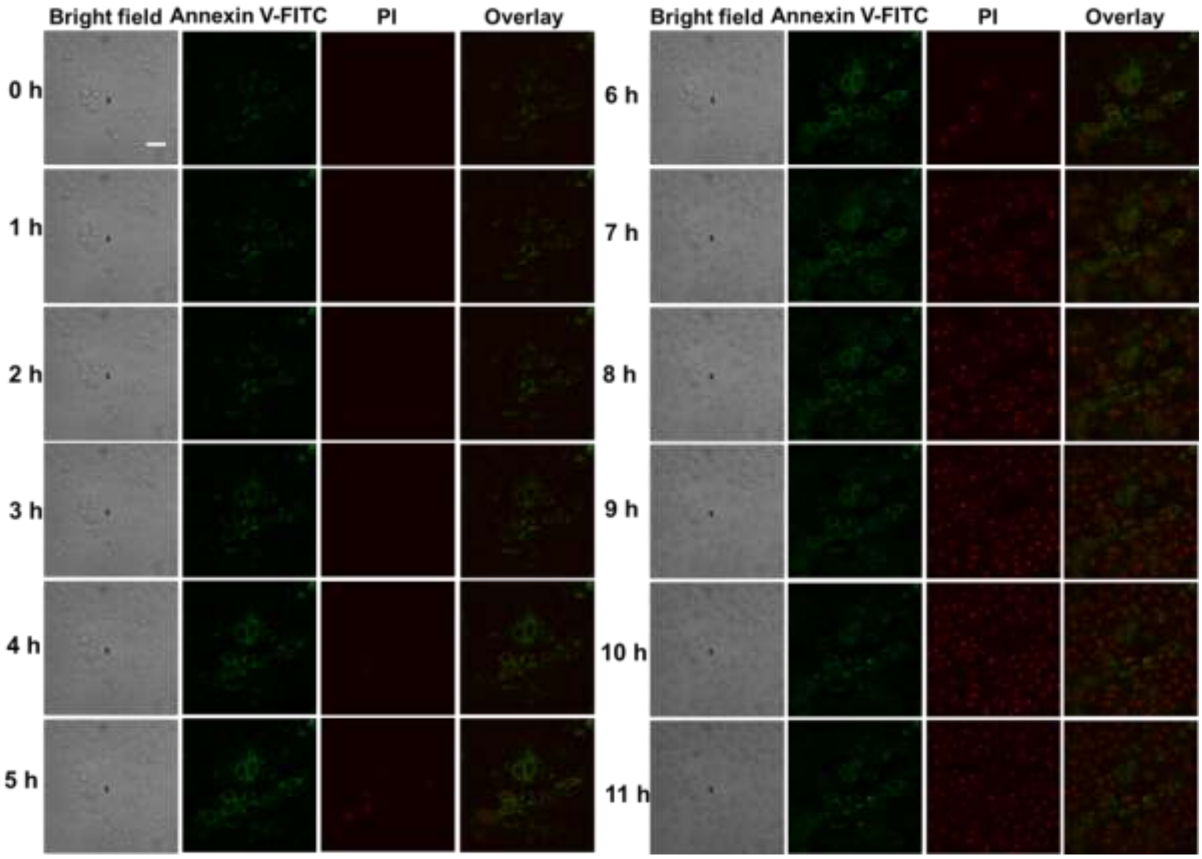

Figure S13. Time-lapse photoluminescence images of Annexin V-FITC/PI stained HepG2 cells after Pdots + NAC co-incubation and light irradiation for 30 min. Scale bars: 40 μm. λ_{ex} = 488 nm.

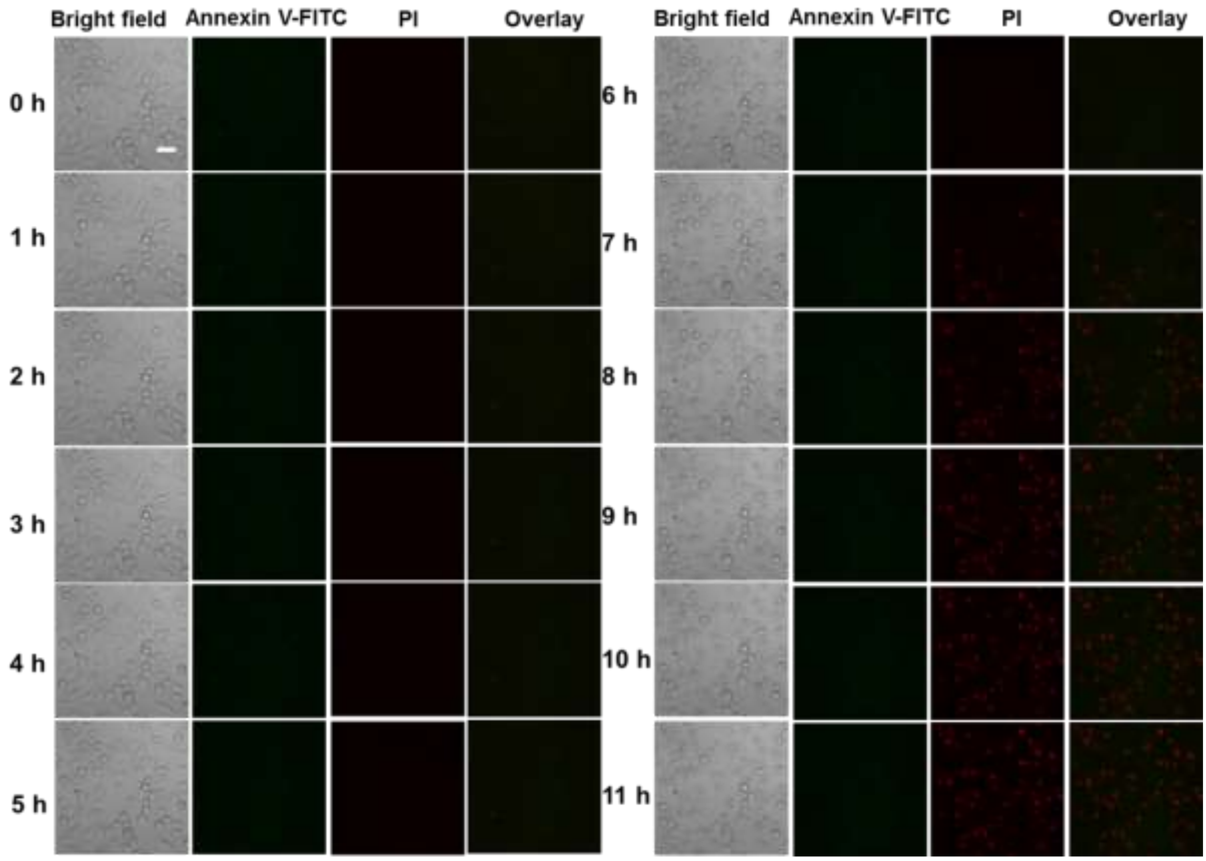

Figure S14. Time-lapse photoluminescence images of Annexin V-FITC/PI stained HepG2 cells after light irradiation for 30 min, but without Pdots incubation. Scale bars: 40 μm. λ_{ex} = 488 nm.

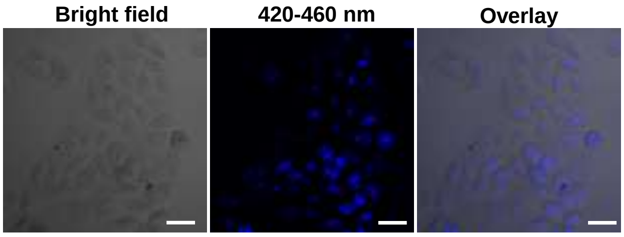

Figure S15. Photoluminescence images of HepG2 cells incubated with Pdots and without light irradiation. Scale bars: 40 μm. λ_{ex} = 405 nm.

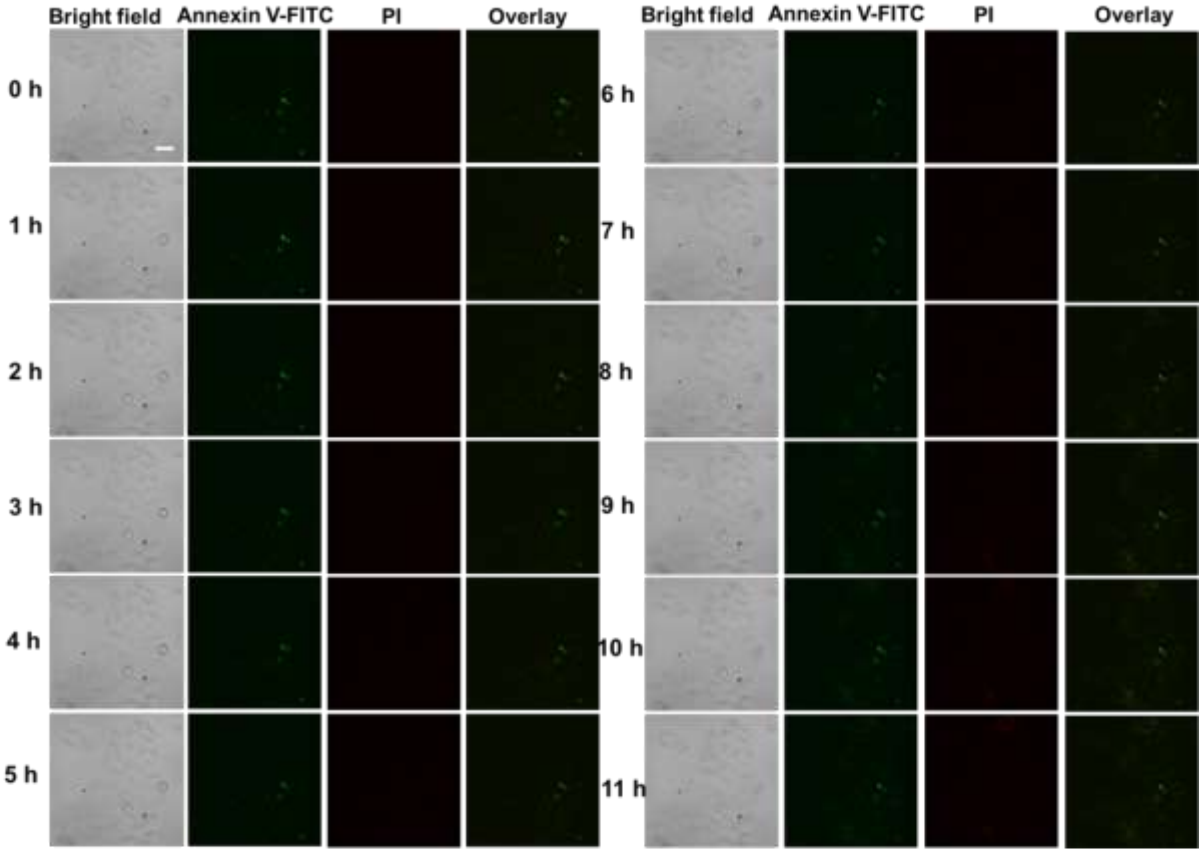

Figure S16. Time-lapse photoluminescence images of Annexin V-FITC/PI stained HepG2 cells after Pdots incubation, but without light irradiation. Scale bars: 40 μm. λ_{ex} = 488 nm.

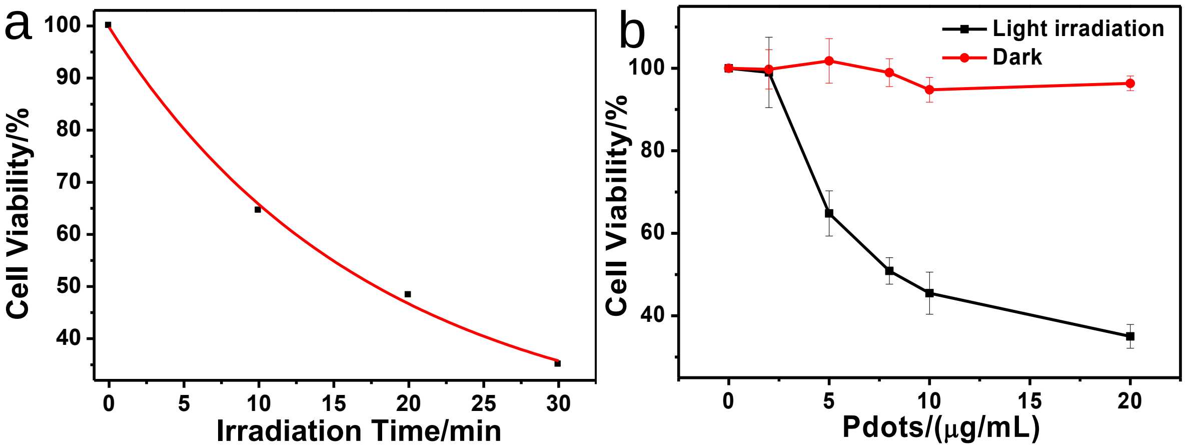

Figure S17. (a) The cell viability of HepG2 cell treated with [Pdots] = 20 μg versus different durations of light exposure. (d) Dose-response curves for cell viability of HepG2 cells treated with Pdots by using a typical MTT assay under light irradiation or in the dark. Error bars correspond to standard deviations from three separate measurements.

Calculation of the Förster radius (R_0):[S1]

The Förster radius was calculated as follows: $R_0^6 = \frac{9k^2\varphi_d J(\lambda)}{128N\pi^5 n^4}$

Where k^2 is the orientation factor of the interacting dipoles (set as 2/3), φ_d the quantum yield of the donor, n the refractive index of the medium separating donor and acceptor chromophore (1.8), $J(\lambda)$ the overlap integral and N the Avogadro's constant.

(S1) C. Wu, D. T. Chiu, *Angew. Chem. Int. Ed.* **2013**, *52*, 3086.
(S2) C. Wu, C. Szymanski, Z. Cain, J. McNeill. *J. Am. Chem. Soc.* **2007**, *129*, 12904.
